# Ecosystem Health Assessment at County-Scale Using the Pressure-State-Response Framework on the Loess Plateau, China

**DOI:** 10.3390/ijerph14010002

**Published:** 2016-12-22

**Authors:** Delin Liu, Shilong Hao

**Affiliations:** 1 Safety and Emergency Management Research Center, Henan Polytechnic University, Jiaozuo 454000, China; 2 Emergency Management School, Henan Polytechnic University, Jiaozuo 454000, China; 3 School of Resources and Environment, North China University of Water Resources and Electric Power, Zhengzhou 450011, China; haoshilong@ncwu.edu.cn; 4 Key Laboratory of the Loess Plateau Soil Erosion and Water Loss Process and Control, Ministry of Water Resources, Yangling 712100, China

**Keywords:** ecosystem health, sustainability, comprehensive indicators, GIS

## Abstract

Assessing ecosystem health is helpful to determine reasonable eco-environmental restoration and resource management strategies. Based on a pressure-state-response (PSR) framework, a set of comprehensive indicators including natural, social and economic aspects was proposed and applied for assessing the ecosystem health of Yuanzhou County, Loess Plateau, Ningxia Province, China. The basic data used to calculate the values of the assessment indicators include Landsat TM image and socio-economic data, and remote sensing (RS) and the geographic information system (GIS) were used to process image data. The results showed that the ecosystem health conditions of most townships in Yuanzhou County were at the moderately healthy level, three townships were at the healthy level, and only two townships were at the unhelathy level; the areas (percentage) at the unhealthy, moderately healthy and healthy levels were 443.91 km^2^ (12.66%), 2438.75 km^2^ (69.54%) and 624.50 km^2^ (17.81%), respectively. The results could provide useful information for local residents and the government to take measures to improve the health conditions of their township ecosystem.

## 1. Introduction

The notion of “health” originated from the field of medical science and was extended to include land by identifying indicators of “land sickness” [1]. In the mid-1980s, this notion was further extended to describe regional ecosystems [2], and the extension resulted in a new concept, ecosystem health, which provides new opportunities to integrate the social, natural and health sciences [3]. Since then, the term “ecosystem health” has been found with increasing frequency in the literature, and an increasing number of environmental managers have begun to consider ecosystem health as a new paradigm for ecological assessment [4,5]. However, due to the complexity of an ecosystem, it is very difficult to make an operational definition and to find a uniform index system to evaluate the health conditions of ecosystems [6]. During the last two decades, a number of different definitions have been proposed and a series of various indicators have been developed for assessing ecosystem health [5,7,8]. Subsequently, many studies have been done to evaluate the health conditions of different aquatic and terrestrial ecosystems, such as coastal ecosystems [9], river/stream ecosystems [10], coral ecosystems [11], agriculture ecosystems [12], soil ecosystems [13] and forest ecosystems [14]. The previous studies provided a theoretical basis and some methods for assessing ecosystem health.

**Figure 1:**
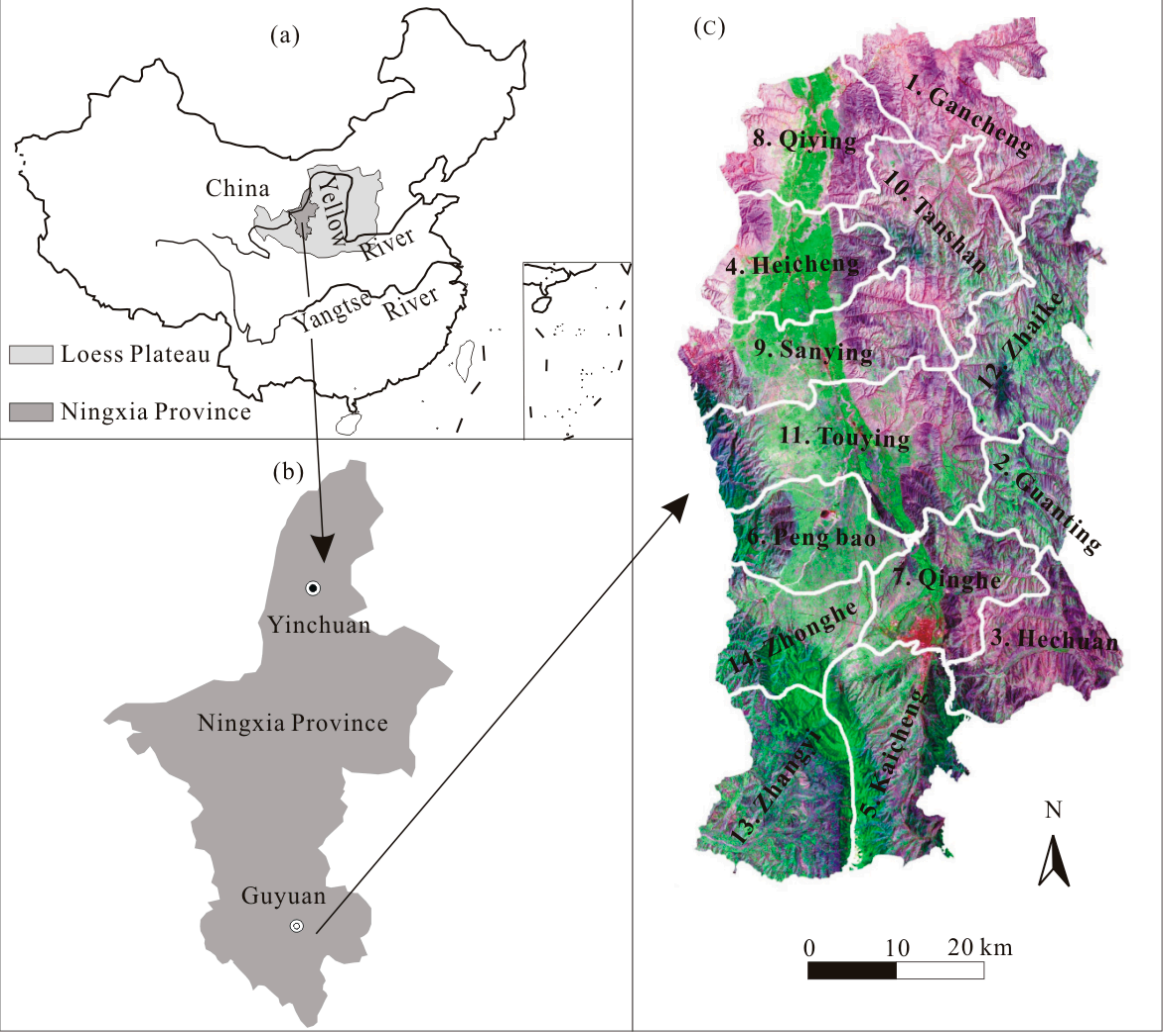
(**a**) Location of Ningxia Province and the Loess Plateau in China; (**b**) Location of the study area in Loess area, Ningxia Province; (**c**) Landsat TM image map of the Yuanzhou County in 2013.

The Loess Plateau, located in the upper and middle reaches of the Yellow River (Figure 1a), is the most severe soil and water loss area in the world [15]. Additionally, severe soil erosion in this region has resulted in a series of eco-environmental problems (mainly including degradation of ecosystem functions, loss of topsoil and agricultural productivity, food security, pollution and sedimentation of downstream rivers, etc.). Fortunately, many researchers have studied these problems from different aspects, and have made a necessary contribution to the eco-environmental restoration on the Loess Plateau [16,17,18]. However, there is very limited literature to study the problems from the point of view of ecosystem health.

A county, the basic administrative unit in China, is considered as the best scale for management and planning of land use [19]. Meanwhile, it is relatively convenient to obtain the socio-economic data of a county. Therefore, the main objective of this paper is, taking Yuanzhou County as an example, to identify a proper method and the corresponding appropriate indicators to assess the health conditions at the county scale on the Loess Plateau, China. The method provides a reliable basis to determine reasonable eco-environmental restoration and resource management strategies for sustainable development.

## 2. Methodology and Case Study

### 2.1. Study Area

Yuanzhou County (35°47’ N–36°38′ N, 105°58′ E–106°32′ E) is located in the southern part of Ningxia Province, a hilly and gullied region of the west Loess Plateau in China, and consists of 14 townships (Figure 1b,c). Its altitude ranges between 1470 and 2900 m above sea level. The total area is 3507 km^2^ in which the mountainous region accounts for about 79.5%. Climatically, it belongs to a semiarid monsoonal region, the annual mean temperature is about 6.2 °C, and the annual mean precipitation is about 471.2 mm. Soil erosion in this region is very severe due to its coarse textured Quaternary loess, long-term degraded forest and the monsoonal conditions. The areas of soil erosion account for greater than 75% and the erosion modulus fluctuates between 3000 and 7800 t/(km^2^·a).

### 2.2. The Pressure-State-Response (PSR) Framework

The PSR framework, proposed by the Organization for Economic Co-Operation and Development [20], was initially developed to provide support for environmental policy-making [21], and now it is used worldwide to study the ecosystem health conditions [22,23,24,25]. For example, Sun et al. (2016) assessed the health conditions of a wetland ecosystem (Hangzhou Bay, China) through integrating remote sensing and statistical data with the PSR framework [22]. Hughey et al. (2004) studied people’s perceptions of the state of the New Zealand environment using the PSR framework [23]. Based on the PSR framework, Ou et al. (2010) developed a sustainable indicator system for local fisheries in Gungliau, Taiwan, between 1995 and 2003 [25]. The PSR framework consist of three categories of indicators, namely pressure indicators, state indicators and response indicators [26]. Here, pressure indicators describe the pressures on ecosystem health exerted by human activities, including resource pressures and social pressures. State indicators reflect the status quo of the ecosystem health, such as the vigor, organization and resilience of an ecosystem. Response indicators show the response degree to the changes of ecosystem health conditions, including changes from humans and the ecosystem itself. The advantages of the PSR framework are (1) to provide a good method to select and organize indicators and data; (2) to help policy-makers design and carry out corresponding strategies [27]; and (3) making health conditions among different ecosystems are comparable because the same assessment index system is used [28]. There are four steps for using the PSR framework to assess ecosystem health conditions as follows.

#### 2.2.1. First Step: Construction of Index System

The PSR framework was suitable for defining assessment indicators because it provides a good method to select and organize indicators and data. Based on the principles of integrity, simplicity and dynamic response [29], nine indicators were selected to build the index system of ecosystem health assessment at the county scale (Table 1).

#### 2.2.2. Second Step: Collection and Processing of Data

The basic data for the analysis mainly include the Landsat TM images with a resolution of 30 m in 2013, the administrative division map of Yuanzhou County in 2003, the topographic map at the scale of 1:50,000, the socio-economic data obtained from the Guyuan yearbook in 2014 [30], and the land use data interpreted from the Landsat TM images. The data were processed as in the following steps. Firstly, the topographic map was taken as the basic map to make geometric corrections for the Landsat TM image and the administrative division map, and then they were converted to the Albers projection (Krassovsky ellipsoid with central meridian 105°, first standard parallel 25° N, second standard parallel 47° N, false easting 0, false northing 0, latitude of projection’s origin 0). Secondly, color composites were generated displaying bands 5, 4 and 3 as red, green and blue, respectively. An image enhancement was performed to increase the visual distinction between different land covers. Thirdly, Yuanzhou County was divided into 14 townships according the administrative division map. Lastly, we obtained the land use types using the visual interpretation of Landsat TM images. If some land use types were not determined from the Landsat TM images, we would carry out a field survey. The field survey method in this paper meant that we went to the field to confirm the land use types in this area. It did not involve national parks, protected areas, endangered or protected species. In order to reduce the possible errors generated in map processing, all the tasks were done by the same person, and the land use classification system with a level 1 class was applied, which only included seven types: arable land, woodland, grassland, orchard, residential land, water body and unutilized land.

Due to the different sources of data, indicators are quite different in categories and their units are also diverse. So, it is necessary to transform all the data into standardized data according to their contributions to the ecosystem’s health. Here, the following two equations are used:

(1) $$X_{i}^{'}=\frac{X_{i}-\min(X_{i})}{\max(X_{i})-\min(X_{i})}\times 100$$

(2) $$X_{i}^{'}=\frac{\max(X_{i})-X_{i}}{\max(X_{i})-\min(X_{i})}\times 100$$

Above, *i* is the number of indicators (here in Equation (1) *i* = 1, 4, 5, 6, 8, 9, and in Equation (2) *i* = 2, 3, 7); X*_i_* and $X_{i}^{\prime }$ are the raw data and standardized data of indicator *i*, respectively, and $X_{i}^{\prime }$∈ (0,100). When the contributions of indicators to ecosystem health are positive, Equation (1) is used, and when they are negative, Equation (2) is used. The standardized data values of each township ecosystem are listed in Table 2.

#### 2.2.3. Third Step: Determination of Index Weights

Several methods can be used to determine the index weights, such as the principle component analysis (PCA) method, the analytic hierarchy process (AHP) method, the entropy method [31], the expert scoring method, and the Delphi method, etc. Each method has its advantages and disadvantages. Here, the weight coefficients (W) of each index were determined by the Delphi method (Table 1).

#### 2.2.4. Fourth Step: Calculation of the Health Index

The following model (Equation (3)) is used to calculate the health index (*HI*) using the processed data and calculated index weights.

(3) $$HI=PI\times W_{P}+SI\times W_{S}+RI\times W_{R}$$

Here, *PI, SI* and *RI* are the pressure index, state index and response index, respectively, and *PI, SI, RI* ∈ (0,100); *W_P_*, *W_S_* and *W_R_* are the weight coefficients of *PI, SI* and *RI*, respectively.

In this paper, the calculated formulae of *PI*, *SI* and *EI* are as follows:

(4) $$PI=\sum_{i=1}^{n}W_{i}\times X_{i}^{'}\;(n\;=\;1,\;2,\;3)$$

(5) $$SI=\sum_{i=4}^{n}W_{i}\times X_{i}^{'}\;(n\;=\;4,\;5,\;6)$$

(6) $$RI=\sum_{i=7}^{n}W_{i}\times X_{i}^{'}\;(n\;=\;7,\;8,\;9)$$

In Equations (4)–(6), n is the scope of the assessment index; *W_i_* and $X_{i}^{\prime }$ are the weight coefficients and standardized data values of indicator *i*, respectively.

According to the standardized data of indicators listed in Table 2, the assessment scores were calculated using the above formulae (Table 3). Based on the assessment scores, three grades are divided using the mean value (MV) and standard deviation (SD) of the assessment scores (Table 3). If the assessment score was lower than 1 SD from the MV (Assessment score < (MV − 1 SD)), the township was in unhealthy level, if the assessment score was greater than 1 SD from MV (Assessment score > (MV + 1 SD)), the township was at a healthy level, and other assessment scores were at moderate healthy levels [28]. The MV and SD of *PI*, *SI*, *RI* and *HI* were also shown in Table 3.

## 3. Assessment Results

### 3.1. Ecosystem Pressure Assessment

The results showed that the pressures faced by most township ecosystems were at the moderately healthy level, and the *PI* values ranged from 19.14 to 66.09. The ecosystems of Gancheng, Tanshan and Zhaike (No. 1, No. 10 and No. 12) were at the healthy level, and only the ecosystem of Qinghe (No. 7) was at the unhealthy level (Table 3). According to the combination of different pressure indicators (Table 2), the pressures of different township ecosystems can be divided into four types. Namely, (1) only one indicator was at the unhealthy level, including the township ecosystems of No. 2, No. 3 and No. 8; (2) both indicators were at the unhealthy level, including the township ecosystems of No. 4, No. 5, No. 11, No. 13 and No. 14; (3) three indicators were at the unhealthy level, including the township ecosystems of No. 6, No. 7 and No. 7; and (4) three indicators were at the healthy level, including the township ecosystems of No. 1, No. 10 and No. 12.

### 3.2. Ecosystem State Assessment

Similar to the results of the pressure assessment, the states of most township ecosystems were at the moderately healthy level, and the *SI* values ranged between 38.69 and 67.84. Only two townships (No. 12 and No. 14) were at the healthy level, and three townships (No. 1, No. 4 and No. 10) were at the unhealthy level. Further analysis of the single indicator (Table 2) revealed that the ecosystem health of Heicheng Township (No. 4) was at the unhealthy level owing to its lower organization and lowest vigor and resilience; Gancheng Township (No. 1) and Tanshan Township (No. 10) were at the unhealthy level due to their three state indicators being lower or the lowest.

### 3.3. Ecosystem Response Assessment

Ecosystems will make the corresponding changes when they are subjected to disturbances or pressure. The *RI* values (Table 3) showed that Qiying (No. 8) was at the unhealthy level, and Guanting and Hechuan (No. 2 and No. 3) were at the healthy level. The other 11 township ecosystems were at the moderately healthy level, and the *SI* values ranged from 40.54 to 72.42. According to Table 2, the main reasons for the unhealthy level in Qiying (No. 8) were its lowest MPS (landscape fragmentation index) and lower income of labor service export.

### 3.4. Integrated Health Assessment of Ecosystem

Ecosystem health is a comprehensive reflection of the pressure, state and response of an ecosystem. It is not only the result of the long-term effect of natural and social factors, but the basic embodiment of ecosystem characteristics and functions. Integrated health assessment results in Table 3 showed that the health conditions of most township ecosystems were at the moderately healthy level. Heicheng (No. 4) and Qingying (No. 8) were at the unhealthy level. Guanting (No. 2), Zhaike (No. 12) and Zhonghe (No. 14) were at the healthy level. The areas (percentage) of Yuanzhou County at the unhealthy, moderate healthy and healthy levels were 443.91 km^2^ (12.66%), 2438.75 km^2^ (69.54%) and 624.50 km^2^ (17.81%), respectively.

## 4. Discussion

### 4.1. Assessment Model and Index System

A number of models and a series of indicators were proposed and used for the assessment of ecosystem health conditions, but each model is only a major organizing paradigm for the assessment of ecosystem health conditions, and each ecosystem has its own indicators reflecting different aspects of its health status [6,32,33,34,35]. For example, Tang et al. (2015) used physical stressors, chemical stressors, community structure metrics and ecosystem-level eco-exergy indicators to construct an index system for assessing coastal ecosystem health [6]. Liu et al. (2009) proposed an urban ecosystem health index based on emergy theory and chose four major factors (vigor, organizational structure, resilience and function maintenance) to assess the urban ecosystem health of Baotou, China [33]. Zhang et al. (2016) developed a driving force–pressure-state-impact-response-management framework and chose 18 specific indicators to study lake ecosystem health conditions [35]. In view of the fact that the ecosystem health was the result of long-term effects of both natural and social factors, the PSR framework was used to select and organize indicators and data. Nine indicators used here were selected using the PSR framework which included natural, social and economic aspects of the ecosystem. The pressure indicators, expressing the natural resource, population and human activity impacts on an ecosystem, were arable land area per capita, the human disturbance index and the population density index. The state indicators covering the vigor, organization and resilience aspects could reflect the natural attributes of an ecosystem, and the specific indicators were the normalized difference vegetation index (NDVI), landscape diversity index (SHDI) and ecological resilience index (ERI), respectively. The response indicators reflected the response of the natural ecosystem and human society to the pressure and state faced by ecosystems. Three indicators were selected in this part, namely the landscape fragmentation index (MPS), the percentage of labor service export and the percentage of income of the labor service export. Compared with other similar studies [6,33,35], the indicators used in this paper are more comprehensive because they cover natural, social and economic aspects of an ecosystem. Besides, the procedures for using the PSR framework to assess ecosystem health were summarized, namely (1) construction of the index system; (2) collection and processing of data; (3) determination of index weights; and (4) calculation of the health index.

### 4.2. Application of the Assessment Results

The ecosystem health at the county scale on the Loess Plateau was assessed based on the PSR framework and the selected indicators. Using the mean value (MV) and standard deviation (SD) of the assessment scores, the scores of *PI*, *SI*, *RI* and *HI* were divided into three categories, namely unhealthy, moderately healthy and healthy levels. The results showed that the health conditions of most township ecosystems were at the moderately healthy level. Heicheng (No. 4) and Qingying (No. 8) were at the unhealthy level. Guanting (No. 2), Zhaike (No. 12) and Zhonghe (No. 14) were at the healthy level. Some suggestions for improving the ecosystem health for townships were proposed based on their particular circumstances and the combined effects of different factors on each specific township ecosystem.

(1) Where health conditions were at the unhealthy level (No. 4 and No. 8). The service function of such townships could no longer maintain the ecosystem, and the systems were degenerating to a certain extent, owing to the lack of arable land, intense human activities, lower vigor and resilience, an unreasonable system structure and response, etc. Therefore, comprehensive measures should be taken to further improve the health status and service functions of these township ecosystems.

(2) Where health conditions were at the moderately healthy level. Most indicators of such township ecosystems were at the moderate healthy level, so they were steady enough to maintain the townships’ ecosystems. However, the health status of such township ecosystems will be improved if we can find out the specific factors affecting the ecosystem’s health, and take the appropriate measures to reduce their adverse impact. In No. 6, as an example, the main indicators affecting its health conditions were the arable land resources, population and income of labor service export (Table 2). Therefore, increasing the amount of labor service export would be a good way to improve the ecosystem health.

### 4.3. Issues for Further Research

The ecosystem health of Yuanzhou County on the Loess Plateau was assessed using the PSR framework, and some useful information was obtained from the assessement resluts. However, there were still some key issues for further research.

(1) The PSR framework was widely used in the field of ecosystem health assessment because of its advantages. However, it was unclear that whether the evaluation results of the PSR framewoke were consistent with the evaluation results of other models. So, comparative studies on the assessment results between the PSR framework and other models (all the models using the same index system) need further research.

(2) The indicators used here were very applicable and could fully reflect the ecosystem health conditions of the study area, but it was just a case study. It was known that different regions have different natural and social characteristics, so whether the indicators used in this paper were suitable for other regions needs further research.

(3) These issues, such as how to make the assessment results more practical, how to propose practical, feasible and detailed suggestions to improve the ecosystem health conditions based on the assessment results, and how to carry out the suggestions effectively, also need further research.

## 5. Conclusions

Knowing about the ecosystem health conditions is helpful for the government and local residents to carry out reasonable eco-environmental restoration and resource management strategies. In order to assess the ecosystem health conditions, nine indicators were developed using the PSR framework and applied for evaluating the ecosystem health conditions of Yuanzhou County, Loess Plateau, Ningxia Province, China. The findings of this study indicated that the health conditions of most township ecosystems were at the moderately healthy level, and the areas (percentage) of Yuanzhou County at the unhealthy, moderately healthy and healthy levels were 443.91 km^2^ (12.66%), 2438.75 km^2^ (69.54%) and 624.50 km^2^ (17.81%), respectively. Based on the assessment results and their particular circumstances, some suggestions for improving the ecosystem health conditions for the townships were also proposed. Finally, some key issues for further research were proposed, which included the comparability of evaluation results between the PSR framework and other models, the applicability of indicators in other regions, and how to make the assessment results and suggestions more practical.

## Figures and Tables

**Table 1:** Index system of ecosystem health assessment and the weight coefficients of each indicator.

Objective	General Criteria		Secondary Criteria		Indicators			
EHA	**Index**	**Weight**	**Index**	**Weight**	**Index**	**Positive or Negative**	**No.**	**Weight**
	Pressure	0.3	Resources pressure	0.4	Arable land area per capita	Positive	X_1_	1.0
			Social pressure	0.6	Population density index	Negative	X_2_	0.5
					Human disturb index	Negative	X_3_	0.5
	State	0.4	Vigor	0.3	Normalized difference vegetation index (NDVI)	Positive	X_4_	1.0
			Organization	0.4	Landscape diversity index (SHDI)	Positive	X_5_	1.0
			Resilience	0.3	Ecological resilience index (ERI)	Positive	X_6_	1.0
	Response	0.3	Natural ecosystem	0.4	Landscape fragmentation index (MPS)	Negative	X_7_	1.0
			Human activity	0.6	Percentage of labor service export	Positive	X_8_	0.5
					Percentage of income of labor service export	Negative	X_9_	0.5

EHA denotes the abbreviation of the ecosystem health assessment; No. refers to the indicator series; X*_i_* refers to the corresponding index (*i* = 1, 2,…, 9).

**Table 2:** Standardized data of indicators in each township ecosystem of Yuanzhou County in 2013.

Township Name	No.	Pressure Index			Organization Index			Response Index		
		$X_{1}^{\prime }$	$X_{2}^{\prime }$	$X_{3}^{\prime }$	$X_{4}^{\prime }$	$X_{5}^{\prime }$	$X_{6}^{\prime }$	$X_{7}^{\prime }$	$X_{8}^{\prime }$	$X_{9}^{\prime }$
Gancheng	1	100.00	100.00	98.41	33.33	12.63	16.67	29.70	71.43	59.46
Guanting	2	34.72	95.08	99.86	100.00	0.00	66.67	100.00	57.14	67.57
Hechuan	3	31.94	75.49	99.42	53.70	18.95	50.00	77.23	100.00	56.76
Heicheng	4	15.28	2.57	49.20	0.00	38.95	0.00	38.61	71.43	13.51
Kaicheng	5	0.00	25.71	62.23	9.26	89.47	83.33	84.16	0.00	0.00
Pengbao	6	13.89	18.02	39.36	66.67	82.11	50.00	58.42	78.57	37.84
Qinghe	7	1.39	4.64	0.00	59.26	77.89	33.33	79.21	42.86	13.51
Qiying	8	22.22	44.05	59.33	37.04	58.95	16.67	0.00	71.43	16.22
Sanying	9	11.11	17.72	40.23	40.74	82.11	33.33	61.39	64.29	18.92
Tanshan	10	55.56	83.89	94.93	27.78	26.32	16.67	22.77	64.29	100.00
Touying	11	15.28	39.50	58.90	35.19	92.63	83.33	73.27	71.43	35.14
Zhaike	12	52.78	93.02	100.00	64.81	36.84	66.67	78.22	64.29	64.86
Zhangyi	13	0.00	0.00	76.56	5.56	78.95	83.33	64.36	50.00	56.76
Zhonghe	14	9.72	27.98	54.70	40.74	100.00	100.00	71.29	71.43	75.68

No. stands for the name of township; $X_{i}^{\prime }$ refers to the corresponding index (*i* = 1, 2, …, 9).

**Table 3:** Calculated results of the index of the pressure, state, response and health in each township ecosystem in 2013.

Health Conditions	No. 1	No. 2	No. 3	No. 4	No. 5	No. 6	No. 7	No. 8	No. 9	No. 10	No. 11	No. 12	No. 13	No. 14	MV	SD
*PI*	99.60	66.09	59.70	20.58	21.99	21.29	1.85	36.96	20.04	72.48	32.24	74.64	19.14	25.53	40.87	28.40
Level	H	M	M	M	M	M	U	M	M	H	M	H	M	M		
*SI*	20.05	50.00	38.69	15.58	63.57	67.84	58.94	39.69	55.06	23.86	72.61	54.18	58.25	82.22	50.04	20.08
Level	U	M	M	U	M	M	M	M	M	U	H	M	M	H		
*RI*	47.57	81.18	77.80	40.54	42.08	58.31	53.70	21.91	51.49	52.46	63.27	71.40	58.87	72.42	56.64	16.13
Level	M	H	H	M	M	M	M	U	M	M	M	M	M	M		
*HI*	52.17	64.18	56.73	24.57	44.65	51.02	40.24	33.54	43.49	47.03	57.70	65.48	46.70	62.27	49.27	11.76
Level	M	H	M	U	M	M	M	U	M	M	M	H	M	H		

No. stands for the name of township; *PI*, *SI*, *RI* and *HI* are the pressure index, state index, response index, and ecosystem health index, respectively, and *PI*, *SI*, *RI*, *HI* ∈ (0,100); H, M and U indicate that the health conditions of each township are at healthy, moderately healthy and unhealthy levels, respectively. MV and SD refer to the mean value and standard deviation of the assessment scores, respectively.
